# The Economics of Fiscal Rules and Debt Sustainability

**DOI:** 10.1007/s10272-022-1021-1

**Published:** 2022-02-12

**Authors:** Roel Beetsma

**Affiliations:** Amsterdam School of Economics, University of Amsterdam, Roetersstraat 11, 1018 WB Amsterdam, Netherlands

**Keywords:** E62, H6

## Abstract

Because they exert cross-border spillover effects, fiscal policies of individual EU member states are a common concern for the entire EU.

Because they exert cross-border spillover effects, fiscal policies of individual EU member states are a common concern for the entire EU. An expansionary fiscal stance in one country raises imports from other countries, thereby stimulating their economies (Beetsma et al., 2006; Alcidi et al., 2015). But it also pushes up the country's public debt and magnifies solvency risk, which may spill over to other member states or force them to come to the financial rescue. These spillovers provide the main rationale for the EU's Stability and Growth Pact (SGP), the most visible elements of which are the 3% of GDP reference value for the deficit and the 60% reference value for the public debt.[1] The SGP strengthens the EU Treaty's "no-bailout" clause by which countries or EU institutions are forbidden to bail out a country in financial difficulty. The rationale behind the clause is that a credible no-bailout policy limits moral hazard on the side of governments. Knowing that no other party may come to the rescue, they will behave responsibly; otherwise, financial markets will force them to do so. In effect, the SGP is the answer to the fear that markets cannot adequately fulfil this role, creating a risk that the no-bailout clause will be tested, which is exactly what has happened.

## The advice of the European Fiscal Board

In the year before the eruption of the coronavirus crisis, the European Fiscal Board (EFB, 2019) offered President Juncker of the European Commission advice. It concluded that high debt ratios had not been sufficiently reduced, especially in periods when this was opportune; that national fiscal policies were too often procyclical; and that the flexibility in the rules had not prevented governments from cutting back on public investment or, more broadly, growth-friendly spending. Failure to take advantage of good times by building buffers resulted in unwarranted budgetary contraction during bad times, the most pronounced example being the period 2011-2013, when countries recorded large improvements in their structural balance at a time of highly negative output gaps. Expenditure slippages went into higher current spending, not into investment. The SGP had numerous ailments: (i) rules were complex and opaque, based on unobservable indicators, while the use of multiple indicators allowed cherry-picking so as to give countries the benefit of the doubt when needed; (ii) medium-term planning was weak, while planned adjustment was back-loaded; and (iii) political considerations interfered with economic assessment, while surveillance was becoming increasingly bilateral between the Commission and the country surveyed.

The EFB (2019) essentially reiterated the revision proposed in its Annual Report 2018 (EFB, 2018). First, the EFB (2019) suggested imposing one fiscal anchor, a debt ceiling at 60% of GDP: the focus would be on sustainability, while its advantage would be its simplicity and observability. Second, it recommended the creation of an expenditure benchmark as a single operating indicator, which is under the control of the government, imposing a ceiling on primary expenditure growth equal to potential output growth,[2] with a correction factor to bring excessive debt down to 60% (in 15 years).[3] This would create a built-in stabilising effect: In periods with actual growth below potential growth, spending growth would exceed actual GDP growth, thereby providing economic stimulus and vice versa in periods with actual growth exceeding potential growth. The spending ceiling would be fixed over the coming three years, after which it would be recalculated. This medium-term orientation would avoid un-

1 See Buti and Gaspar (2021) on the history of these reference values. In the following, we will refer to these as "ceilings", although they are not absolute ceilings that can never be exceeded.

* The author thanks Michel Heijdra for comments on an earlier version of this paper. The views in this paper are the author's personal views and should not be attributed to any institution he is affiliated with.

**Roel Beetsma**, University of Amsterdam, Netherlands; and European Fiscal Board, Brussels, Belgium.

2 As is the case for the output gap and the structural balance, potential output growth is not directly observable either. Measurement error is smaller because by taking growth rates, measurement error in the assessment of the level of potential output largely washes out. Claeys et al. (2016) point to the smaller revision errors in medium-term potential growth estimates when compared to changes in the structural balance. However, Barnes and Casey (2019) demonstrate a positive pass-through from revisions in actual to revisions in potential output, which could lead to pro-cyclicality of a spending rule linked to potential output growth.

3 Long-term debt anchors in combination with an intermediate spending ceiling have been proposed by others as well, for example Bénassy-Quéré et al. (2018) and Darvas et al. (2018). The ceiling would correct for discretionary revenue measures and cyclical spending (on unemployment benefits). This way the ceiling gives room to the automatic stabilisers on both the spending and revenue side. A ceiling based on nominal growth would allow additional room for stabilisation if demand shocks dominate: when demand is low, actual inflation undershoots its forecast and spending is allowed to grow even faster relative to actual output.

due policy fluctuations. Third, the EFB recommended introducing a single escape clause replacing all existing flexibility provisions. This would do away with the current "complete contract" approach. And finally, it suggested demarcating policy decisions from economic analysis. The escape clause would be triggered by an independent analysis leading to independent advice that decision-makers at the political level would either follow or deviate from with a motivation.

## The COVID-19 crisis has strengthened the case for reform

The coronavirus crisis has led to a jump in public debt ratios, as a result of the operation of the automatic stabilisers, large-scale discretionary measures and a drop in GDP. Countries with the highest debt ratios before the COVID-19 pandemic recorded on average the largest increases – see Figure 1. The SGP's severe economic downturn (SED) clause (the "general escape clause" in popular terms) was activated to allow for additional flexibility in the application of the pact. No excessive deficit procedures were opened, even though they could have provided some guidance for fiscal policy.

The crisis has made a revision of the SGP even more urgent. Ideally, the time before the deactivation of the SED clause would be used to design a reform of the pact and get countries to agree on the reform, a position also taken by EU Independent Fiscal Institutions (2021).[4] However, due to the expectation that the SED clause will be lifted at the end of 2022 and the fact that countries will need to prepare their budgets for 2023 in the fall of 2022, this would be close to impossible, realistically speaking. Yet, following the Commission consultation, there might be time to produce a blueprint for a revision, which would orient the Commission on how it could apply the pact during the transition to a revised arrangement. This position is not shared by all stakeholders, however. In a recent position paper (Blümel et al., 2021), eight finance ministers indicate that a possible reform of the SGP should not be linked to the de-activation of the SED clause. Nevertheless, they write that they are

> open to a debate on improving economic and fiscal governance, including the Stability and Growth Pact. While sticking to a rules-based fiscal framework, improvements should be made. In particular, simplifications and adaptations that favour consistent, transparent and better application as well as enforcement of the rules are worth discussing, but only if new proposals do not jeopardise the fiscal sustainability of Member States, the Euro Area or the Union as a whole. (Blümel et al., 2021)

4 Martin et al. (2021) go further. They propose conditioning the deactivation of the SED clause on countries that have reached an agreement on a revision of the EU fiscal framework.

Figure 1
**Debt developments by country group**

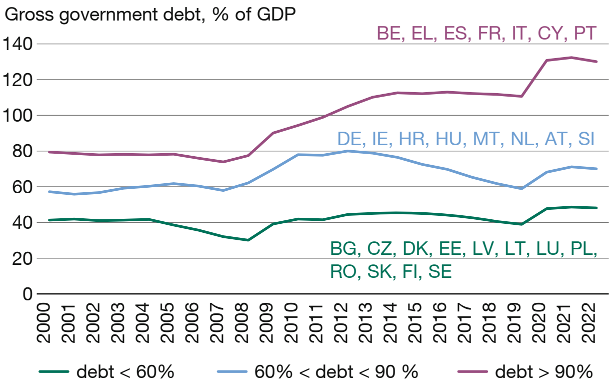

Note: Countries are grouped based on their average debt levels in 2011-2019.

Source: European Fiscal Board.

## Modifying debt requirements

Sustainability should remain the main objective of the SGP. However, the reality is that the debt ratios of several countries are well above 100% of GDP and, hence, the current 60% ceiling remains unattainable for a long period to come. It seems politically impossible to ask countries to run a structural primary surplus of more than 3%-4% for a decade, as numerical analysis by the European Fiscal Board (2018) for some countries suggests is required for a situation more benign than the current one.[5] Regardless, these countries would remain well above 60% after 15 years. The coronavirus crisis has only worsened the debt reduction burden.

Calls for doing away with the current 60% debt reference value and allowing for much higher debt levels come as no surprise. These calls are motivated by the current low nominal interest rates and the expectation, in the financial markets, that interest rates will remain low long into the future. Allowing for higher debt would reduce the pressure for harmful consolidation and enable governments to make the necessary investments in the energy transition and the digitalisation of their economies.

However, this is one side of the debate. There are sensible counterarguments. First, financial markets tend to be short-sighted and may prove to be wrong in their assessment of future interest rates. Inflation has gone up sharply recently. While this may not be the baseline scenario, there is a

5 Eichengreen and Panizza (2014) show that such long periods of fiscal restraint are historically unprecedented. Note that the analysis was done under specific macroeconomic assumptions, such as a gradual increase in the real interest rate and no feedback effect from the level of debt to the risk premium on the debt. Doing away with these assumptions speeds up the debt reduction considerably.

non-negligible chance that inflation will remain elevated in the face of continued supply constraints, high demand and a shortage of labour that pushes wages up. Moreover, the current loose monetary policy conditions affect inflation only with considerable lag, so they may cause more inflation in the medium run than we foresee now. A worldwide increase in investment in the climate transition and digitalisation may shift the savings-investment balance, leading to a rise in long interest rates as well. Higher debt levels increase the sensitivity of government finances to rising interest rates. The speed of the pass-through obviously depends on the debt maturity time profile. Second, new major crises may occur.[6] The three crises since the turn of the century were largely unforeseen. A new crisis in the coming decade is more than a theoretical possibility. Third, the costs of the energy transition and climate-related disasters may turn out to be far higher than anticipated. All these arguments speak in favour of a conservative approach to public debt.

Despite these arguments, the reality of the extremely high debt levels may force deviations from the previously agreed upon public debt in the SGP. Adherence to the one-twentieth rule may not immediately be problematic, as the pick-up of growth when coming out of the coronavirus crisis will exert a strong negative effect on debt ratios via the so-called "snowball effect".[7] However, the rule will likely be constraining further down the road. Box 1 describes some possible scenarios for allowing milder debt reduction trajectories of (very) high debt countries. However, an alleviation of debt reduction requirements begs the crucial question: If countries did not adhere to the required debt reduction in the past, how could we get them to adhere to a milder path now? On the one hand, it can be argued that imposing softer, but more realistic, requirements makes these more credible. On the other hand, a softening now raises expectations of new revisions in the future.

A relaxation of debt reduction requirements would need a revision of the SGP also in other dimensions to enhance the credibility of the debt reduction strategies. While we cannot expect perfect adherence to new reduction paths, an appropriate revision can encourage governments to improve their behaviour. Under the EFB proposals, the rules will be simplified and less reliant on unobservable variables. Use of the escape clause will be better justified on economic grounds. Hence, it will become harder to justify not undertaking the required surveillance actions when fiscal requirements are violated.

6 Using the example of Sweden, Andersson and Jonung (2019) show that a banking crisis can easily add an extra 25-30 percentage points to the existing debt ratio of GDP.

7 This effect is the difference between nominal GDP growth and the average nominal interest rate on the debt multiplied by the debt ratio. The debt ratio falls "automatically" if the aforementioned difference is positive.

Box 1

**Setting debt reduction requirement**

Economic theory cannot answer the question about what is *the* optimal public debt level. The optimal debt level at each moment in the future will depend on a number of factors: the initial debt level; the projected distribution over time of spending needs (on pensions, healthcare, energy transition, etc.); the political weight attached to the different current and future cohorts; the risk of insolvency; and, in the EU context, what is politically achievable within a common fiscal framework. Therefore, any decision on a debt ceiling in the EU will involve a certain degree of arbitrariness and undesirable lack of flexibility.

The reality of extremely high debt levels in the EU could be dealt with in one of four ways. The first method is to not touch the rules but rather accept more transgressions of the rules. Secondly, one could differentiate adjustment speeds towards the current common 60% ceiling (see EFB, 2020). A third possibility would be to raise the debt ceiling for all countries, but leave the adjustment speed of reducing the excess over the ceiling by one-twentieth a year unchanged. A fourth alternative would be to set different ceilings depending on each country's individual situation.

The second and the fourth options can be merged by maintaining the 60% reference value as a very long-run ceiling and agreeing on a differentiated set of debt ceilings at a horizon of, say, seven years, after which a new set of ceilings will be set for the next seven-year period, and so on, with a view to gradually reducing each country's debt to 60% or below. Such an approach may be acceptable to all member states by facing up to the reality that extremely high debt levels can only be reduced gradually by maintaining a sufficiently equitable horizontal treatment. This would require keeping a common, very long-run debt ceiling and not changing the 60% reference value, which would require countries to unanimously agree on a revision of Protocol 12 of the Treaty. It should be noted, though, that revising the one-twentieth rule embedded in Regulation (EC) No 1467/97 may also require unanimity.

Additional measures and provisions should help to instil more credibility. First, the national independent fiscal institutions (IFIs) could be given a larger role in monitoring national debt developments (see Martin et al., 2021). Second, governments could be encouraged to demonstrate their commitment to a revised set of debt requirements by orienting their budgetary planning more towards the medium run. For example, the Netherlands has been quite successful in this respect by imposing spending ceilings on individual public sectors at the start of a new government over the entire cabinet period. Third, legal guarantees at the national level constraining indebtedness can be strengthened. Fourth, the credibility of the no-bailout clause could be improved, for example, by installing infrastructure for an orderly sovereign default and by gradually tightening concentration limits on bank balance sheets or introducing and gradually differentiating risk weights on sovereign debt on bank balance sheets.[8] Fifth, a debt reduction fund can be set up that matches public debt reduction with a contribution from the fund. Such support would only be maintained if a country does not lapse back into fiscal profligacy.[9] While introducing any such measures will be politically sensitive, the desire to revise debt requirements when the SED is deactivated might create room for a grander bargain that includes one or more of these measures.[10]

## Protecting public investment

Public investment or, more broadly, growth-friendly public spending suffered following the global financial crisis, especially in very high debt countries (see Figure 2). There is a danger that the same thing will happen under financial pressure in the aftermath of the coronavirus crisis and related political pressure to protect current spending. Hence, there is substantial support for a "golden rule" that would keep public investment out of the deficit calculation relevant for the 3% ceiling. The fact is, though, that the current SGP already admits a flexible treatment of public investment that has been used very little so far, potentially because the conditions are quite onerous. An important issue associated with this type of flexibility is, of course, that governments have an incentive to classify other types of spending as investment spending. Therefore, the EFB has in the past suggested the use of a "modified golden rule" by which, under the condition that debt sustainability is not endangered, investment spending on projects cofinanced and hence vetted by the EU is taken out of the deficit calculation. Potential top-ups by governments of these projects would also be removed. Currently, there is substantial sympathy for a "green golden rule", by which climate investment would be exempt, but it runs the risk of "greenwashing".

Besides classification risks, the need for an integral trade-off on all spending items argues against taking items out of

Figure 2

**Productive public spending as a share of total spending 2017 versus 2007**

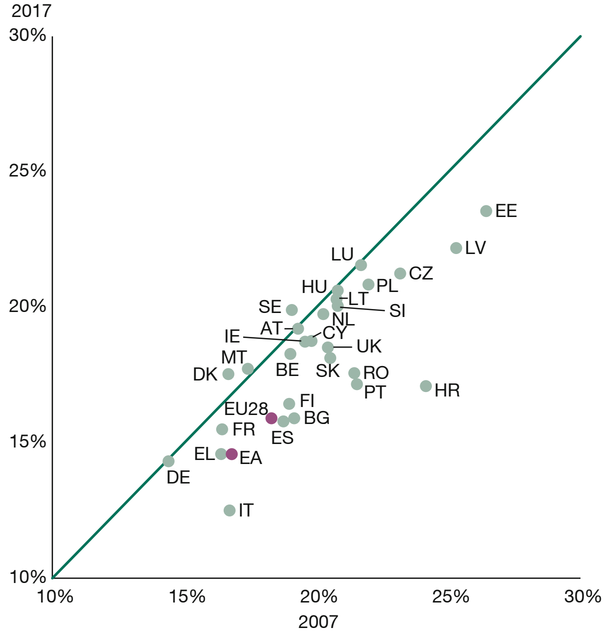

Source: European Fiscal Board.

the calculation of the deficit. An alternative way of stimulating public investment (on climate transition) is to have dedicated national envelopes within the EU budget that countries could spend on public investment. In the case of a failure to use all the dedicated funds, the remainder would flow back into the common part of the EU budget.

## Fiscal standards

Blanchard et al. (2021) bemoan the growing complexity of the SGP and propose abolishing numerical fiscal rules and replacing them with "fiscal standards," which could be enforced by, for example, the European Court of Justice (ECJ). The appeal is to no longer be led by numerology that has little substantive support from economic theory and to focus enforcement on the need to maintain debt sustainability, which would be the relevant fiscal standard. While this approach seems attractive, it has a number of limitations. First, although the precise numbers in the SGP are not justified on good economic grounds, they have nevertheless come to serve as a beacon for budgetary policy, thereby constraining governments in their profligacy. The 3% deficit ceiling is particularly useful in this regard (EFB, 2021). It is highly visible, and the position of the actual deficit relative to this ceiling can be established quite unambiguously. Second, a member state's adherence to the standard will likely be determined ex post, although it is conceivable that a country may be brought before the ECJ based on its plans. However, a ruling takes time and therefore

8 See also European Economy Expert Group (2021). These measures would constrain moral hazard, thereby inducing governments to constrain profligacy.
9 See also European Economy Expert Group (2021).
10 There are various other measures that could be implemented, such as naming and shaming of non-compliant member states, mandatory spending reviews/review frameworks, revenue reviews for countries with a narrow tax base, etc. No single measure will contain the "silver bullet". They may all provide marginal improvements. See also Beetsma and Larch (2018) on the risk-reduction versus risk-sharing debate. The scope for a reform of the EU fiscal architecture is largest if both "camps", i.e. those in favour of risk reduction and those in favour of risk sharing, receive something in return for what they demand.

it will not always be clear whether a proposed policy is in line with the standard. Third, the ECJ will need to build up capacity and case law to establish whether a followed policy is in line with the standard. A large number of cases before the court simultaneously will absorb a great deal of the court's capacity. All in all, there are strong arguments to keep numerical rules.

## A numerical ceiling on interest payments

Even though public debt has risen, debt interest payments have fallen, prompting some experts to argue that it would be preferable to replace the current numerical SGP ceilings with a ceiling on interest expenditures on public debt.[11] This would be risky, however. In an era of very low interest rates, this may encourage countries to run up extremely high debt levels before the ceiling on interest spending is reached, which would pose risks for financial stability if interest rates start rising again.

## The role of the national IFIs

The national IFIs are a very heterogeneous group of institutions with different effective independence, resources, assignments and operational contexts (see e.g. Beetsma et al., 2019). They pay limited attention at best to potential spillovers from national fiscal policy. Hence, EU level fiscal surveillance under a common set of budgetary rules will remain necessary for a well-functioning EU. Still, with their more detailed knowledge of their own country's situation, national IFIs could assume a larger role in monitoring national debt developments, especially when the SGP is revised and debt reduction requirements become more tailor made to the specific situation of individual countries. The IFIs could analyse whether potential violations of the requirements are justified on the basis of developments outside the control of the government and provide the Commission with input for its surveillance actions. In their proposal, Martin et al. (2021) assign a key role to the IFIs. Governments propose a five-year debt target and primary expenditure consistent with the target. The national IFI assesses the sustainability of the public finances, based on a common methodology set by the EFB, and validates the debt target. This serves as input for the Commission which provides a recommendation on the target and spending path, after which the Ecofin accepts or rejects the proposals. Clearly, some IFIs would need to strengthen their analytical capacity under this proposal.

## Concluding remarks

Even though they can never be perfect, numerical fiscal rules are needed to restrain governments in their budgetary policies. As argued, once the SED clause is lifted, it is important to have a blueprint for a revised SGP, because returning to the original surveillance practice when the original rules cannot be adhered to will further undermine the SGP's credibility. Also, the momentum for reform may abate. It is crucial that changes in or differentiation of debt reduction requirements post COVID-19 be accompanied by an enhanced commitment to the revised requirements. Such a commitment will be strengthened by the revision of the SGP in other dimensions, which would make it simpler and more transparent, and by whether national IFIs can assume a larger role in monitoring debt developments.

11 For the US Furman and Summers (2020) argue for capping interest spending to 2% of GDP.
